# Deep breathing in your hands: designing and assessing a DTx mobile app

**DOI:** 10.3389/fdgth.2024.1287340

**Published:** 2024-01-29

**Authors:** Harim Jeong, Joo Hun Yoo, Michelle Goh, Hayeon Song

**Affiliations:** ^1^ Department of Interaction Science, SungKyunKwan University, Seoul, South Korea; ^2^ Department of Artificial Intelligence, SungKyunKwan University, Suwon, South Korea; ^3^ Hippo T&C, Suwon, South Korea

**Keywords:** digital therapeutics (DTx), Human–Computer Interaction (HCI), mobile health interventions, machine learning feedback, gamification design, user engagement

## Abstract

Digital Therapeutics (DTx) are experiencing rapid advancements within mobile and mental healthcare sectors, with their ubiquity and enhanced accessibility setting them apart as uniquely effective solutions. In this evolving context, our research focuses on deep breathing, a vital technique in mental health management, aiming to optimize its application in DTx mobile platforms. Based on well-founded theories, we introduced a gamified and affordance-driven design, facilitating intuitive breath control. To enhance user engagement, we deployed the Mel Frequency Cepstral Coefficient (MFCC)-driven personalized machine learning method for accurate biofeedback visualization. To assess our design, we enlisted 70 participants, segregating them into a control and an intervention group. We evaluated Heart Rate Variability (HRV) metrics and collated user experience feedback. A key finding of our research is the stabilization of the Standard Deviation of the NN Interval (SDNN) within Heart Rate Variability (HRV), which is critical for stress reduction and overall health improvement. Our intervention group observed a pronounced stabilization in SDNN, indicating significant stress alleviation compared to the control group. This finding underscores the practical impact of our DTx solution in managing stress and promoting mental health. Furthermore, in the assessment of our intervention cohort, we observed a significant increase in perceived enjoyment, with a notable 22% higher score and 10.69% increase in positive attitudes toward the application compared to the control group. These metrics underscore our DTx solution’s effectiveness in improving user engagement and fostering a positive disposition toward digital therapeutic efficacy. Although current technology poses challenges in seamlessly incorporating machine learning into mobile platforms, our model demonstrated superior effectiveness and user experience compared to existing solutions. We believe this result demonstrates the potential of our user-centric machine learning techniques, such as gamified and affordance-based approaches with MFCC, which could contribute significantly to the field of mobile mental healthcare.

## 1 Introduction

The digital healthcare and digital therapeutics (DTx) market has been experiencing a rapid expansion, a surge further catalyzed by the COVID-19 pandemic, which underscores the need for digital solutions to address mental health disorders (1). Current research indicates that of 18 identified DTx products, only six specifically target on treating mental disorders with a particular emphasis on depression, anxiety disorders, and insomnia (2, 3). This pattern aligns with the U.S. Food and Drug Administration’s (FDA) temporary policy to broaden patient access to DTx for mental healthcare amidst the pandemic (4).

In the realm of the mental health-centric DTx, the digital implementations of Cognitive Behavioral Therapy (CBT) are prevalent (3), often incorporating breathing exercises into these interventions (5). Notably, the integration of CBT and breathing exercises has been proposed to amplify therapeutic benefits (6). Building on this foundation, our study introduces an innovative approach to breathing exercises, aimed to enhance the effectiveness of an array of DTx targeting mental disorders. We aspire to increase their potency while ensuring sustained usage, a crucial aspect of successful DTx deployment. Our primary research goal is to foster the development of a more proficient DTx by evaluating the effectiveness of our proposed breathing exercise method.

Due to its potency in fostering engagement and elevating motivation, gamification is an active method in learning domains (7). It merges entertainment and tasks by integrating game elements into non-gaming contexts. This technique is especially prevalent in digital healthcare, essential for driving behavior change. In digital health services, where prolonged use is often necessary, gamification enhances engagement and supports sustained user participation. This aligns with the goals of these services. A systematic review demonstrates that gamification and serious games effectively encourage behavior change and heighten motivation (8). These elements contribute to the expectation of improved treatment outcomes within digital health interventions.

At present, DTx targeting mental health are mainly presented as mobile applications (3). Numerous mobile applications focus on deep breathing exercises. Unfortunately, many of these applications primarily offer passive animations and fail to actively engage users, a crucial component for effective DTx and healthcare. Research suggests employing machine learning techniques to offer personalized feedback in such applications (9). Yet, the limited computational prowess of current mobile devices introduces practical challenges. Accordingly, our study presents an efficient system designed to minimize the model weight for smooth operation in a mobile application environment, thus actualizing the methods proposed in prior research.

## 2 Literature review

### 2.1 Gamification effect

Following the broader discussion of gamification’s role in digital healthcare in the introduction, we now focus on its specific impact and application in digital therapeutic interventions. While gamification has been acknowledged for its ability to merge entertainment with tasks, its application in digital health goes beyond mere engagement (10). It plays a significant role in facilitating sustained user interaction, particularly in applications requiring long-term commitment and adherence (11).

In the context of digital therapeutics, gamification is not just about adding game-like elements; it’s about creating a more immersive and interactive experience that resonates with users (12). This approach is crucial in interventions where user motivation and continuous participation are key to successful outcomes. Moreover, gamification strategies in digital health have been shown to effectively drive behavior change, a central goal in many therapeutic interventions (13).

By integrating these elements into digital health interventions, we can transform the user experience from passive to active, thereby potentially improving adherence and treatment outcomes (14). This aligns with the overarching goal of digital therapeutics: to engage users in a meaningful way that promotes positive health behaviors and outcomes. The use of gamification in our study aims to leverage these benefits, creating an engaging and effective platform for deep breathing exercises.

**Figure 1:**
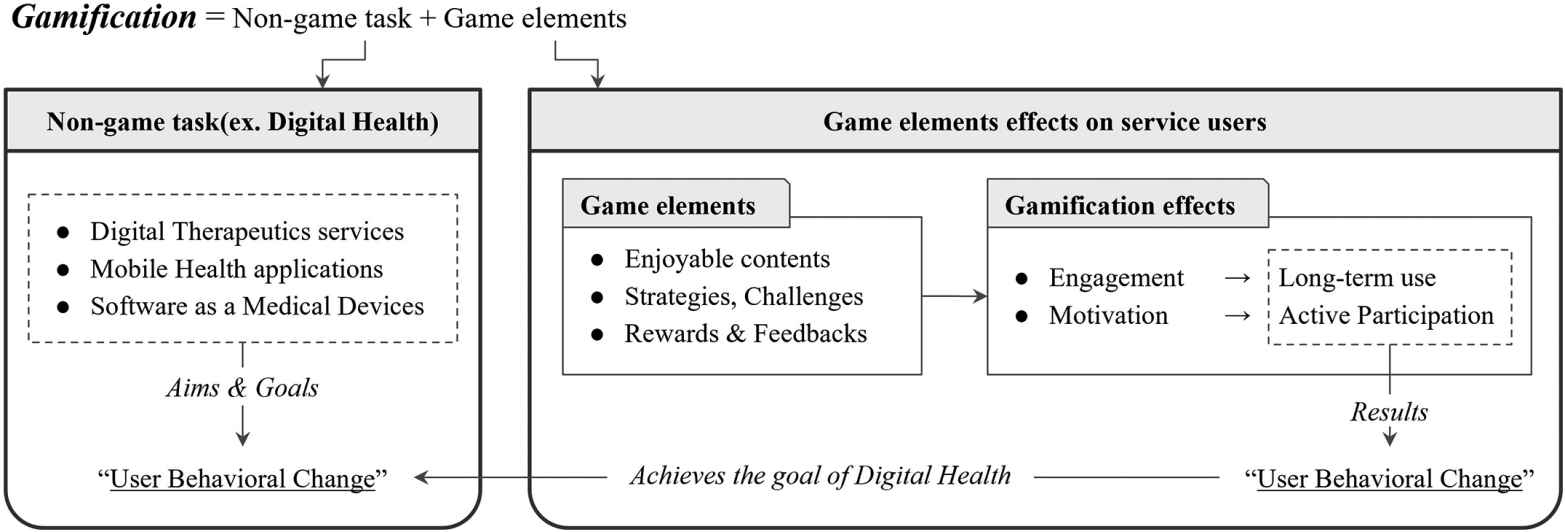
Effects of gamification and its flow.

Based on our review of gamification and its application in healthcare, the effects and flow of gamification have been summarized as follows. This summary illustrates how gamification can transition the user experience from a passive to an active role, underlining its significance in enhancing user engagement and motivation. This transformation is crucial in digital therapeutics, where active participation can lead to improved health outcomes. A visual representation of the effects and flow of gamification, as derived from our review, is depicted in Figure 1 below.

### 2.2 Gamification in deep breathing

As the digital therapeutic landscape continues to evolve, there’s an increasing emphasis on enhancing user engagement to maximize therapeutic benefits. One innovative strategy that’s gaining traction is the incorporation of gamification into deep breathing interventions. By gamifying deep breathing exercises, these interventions aim not only to harness the therapeutic advantages of controlled respiration but also to elevate user commitment, thereby improving the overall effectiveness of the intervention (15, 16).

Building upon the foundation set by previous studies, such as “Calm: Blow away your Stress” and “Breeze”, our research proposes an optimal design for gamifying deep breathing interventions that target relaxation. For instance, the mobile application “Calm: Blow away your Stress” (15) invites users to dissipate twelve clouds using their breath through the microphone. The application’s animated clouds and breath-based feedback stimulate user curiosity and engagement. However, the study did not distinctly address the explicit impact of these elements on engagement.

Another gamified deep breathing intervention is exemplified by “Breeze” (17). In this application, users control the sailing of a boat in a game-like setting by breathing directly into the microphone. The design incorporates a changing background as the boat sails further, encouraging continued user interaction. Feedback is provided based on the user’s control over the boat’s movement, with the Mel-Frequency Cepstral Coefficients (MFCC) feature extraction technique ensuring the feedback’s accuracy by assessing the user’s breathing state.

### 2.3 Affordance-based design

One of the main components of digital health is self-care. This feature signifies a shift from the traditional approach of relying on hospital visits and medical staff for diagnoses and treatments, empowering individuals to manage their own health needs as required (18). Because digital health or DTx requires self-management and system utilization by the user, Human–Computer Interaction (HCI) becomes an essential aspect. Specifically, appropriate guidance should be integrated within the system to ensure users can easily navigate digital health systems (19).

One design element to facilitate this intuitive guidance is affordance-based design. Affordances, defined as the inherent qualities of a design that dictate how an object should be interacted with, can help address a major challenge in digital health: providing comprehensive guidance with minimal user effort (20). Beyond the affordance design for simple self-management, it is posited that for digital health, the effectiveness of treatment can be maximized when the five objectives—social, cognitive, identity, emotional, and functional—are well-communicated through affordances (21).

The paper emphasizes the different affordances’ importance in digital health, which includes social elements fostering a sense of belonging and support, cognitive aspects articulating the service’s advantages, identity elements catering to users’ ideal self-aspirations, emotional components generating positive sentiments, and functional attributes using technology to increase acceptance.

In summary, where self-care is paramount in digital health, an affordance-based design that helps users easily understand how to use the system is crucial. The effect can also be maximized when the five key affordances are adequately fulfilled.

### 2.4 Machine learning for deep breathing

Technological advancements in machine learning have significantly enhanced the domain of relaxation therapy, especially in the application of deep breathing exercises (22). The use of mobile devices’ microphone modules for real-time assessment and feedback of breathing states represents a major leap forward (17). This development is crucial for providing users with immediate and accurate feedback on their breathing patterns, essential for effective relaxation therapy (23).

Machine learning techniques, such as Mel Frequency Cepstral Coefficient (MFCC), have been particularly instrumental in this regard. Originally used in speech recognition (24, 25), MFCC has now become a primary technique for analyzing breathing data. Its ability to extract significant features through spectral analysis has been crucial in developing more sophisticated and user-friendly relaxation therapy applications (26).

Building on this foundation, our study has implemented a deep breathing feature that provides accurate feedback using MFCC. Considering that most mental healthcare software is currently mobile-based, our focus has been on developing a system that is well-suited for the mobile environment. This approach ensures that our deep breathing exercises are not only effective but also accessible and practical for users on mobile platforms, aligning with the trend towards mobile health solutions in mental healthcare.

### 2.5 Heart rate variability (HRV) measurement

Heart Rate Variability (HRV) serves as a major indicator of the autonomic nervous system’s functionality and has gained importance in the study of mental disorders (27). The analysis of HRV data, using methods such as frequency domain and time domain analysis, provides valuable insights into an individual’s physiological responses under different conditions (28). These insights are pivotal for understanding the impact of various therapies, including deep breathing exercises, on mental well-being (29).

The measurement of HRV, therefore, becomes an integral part of our study, offering a window into the physiological impacts of our proposed deep breathing exercises. This rich source of data is instrumental in evaluating the efficacy of our interventions in a quantifiable and scientific manner. Considering the objective and scientific nature of HRV measurement, we have chosen this method as a key component to substantiate the effectiveness of our study. By proving the efficacy through HRV analysis, we aim to provide more objective and concrete evidence of the therapeutic benefits, further validating the practical implications of our research in mental health treatment.

## 3 Methodology

### 3.1 Designing a gamified deep breathing system

The present study introduces a gamified deep breathing system, infusing gamification principles into relaxation exercises. This novel approach aspires to alleviate the monotony of traditional deep breathing exercises, aiming for increased user immersion. To foster continuous engagement, our system offers real-time feedback, employing machine learning to provide accurate insights into the user’s breathing dynamics.

Our design incorporates ’affordance-based design’ principles, facilitating intuitive user perception and interactions within the application environment. The user interface employs straightforward and intuitive on-screen visuals corresponding with different breathing phases: inhalation, exhalation, and rest. During inhalation, a ’wind’ effect descends on the screen, guiding users to breathe in. For exhalation, users blow into the microphone, causing the ’wind’ effect to ascend, reflecting the exhale action. Finally, in the rest phase, the ’wind’ effect disappears, signaling users to pause their breathing. The actual implementation of this breathing system can be seen in Figure 3.

In contrast, the application used by the control group, while also providing visual cues for breathing indication, lacked the gamification and affordance design elements of our system. This key difference lies in the absence of interactive elements such as the ’wind’ effect and the dandelion seed metaphor, which are integral to our system’s user engagement strategy. The control group’s application primarily relied on simpler visual cues without the interactive and immersive components that characterize our gamified approach. An example screen of the application used by the control group can be found in Figure 4.

This design approach ensures a more engaging and interactive experience, aligning with our goal of enhancing user immersion in deep breathing exercises through gamification. By visually and interactively guiding users through each breathing phase, the system provides a unique and effective way to practice deep breathing, making the exercise both enjoyable and beneficial.

A pivotal design feature is the dandelion seed, a visual metaphor symbolizing the need for gentle, controlled breathing. Considering the key affordances of digital health outlined by Wong et al. (21), our design integrates elements of cognitive, emotional, identity, and functional affordances. The social aspect, though ideal, is outside this study’s scope. For cognitive reinforcement, the application elucidates the merits of deep breathing and its mental health impacts. Positive feedback messages uplift emotional affordance. The system’s visualization of a dandelion ascending and ultimately blossoming reinforces identity. Clear insights into the system’s workings (e.g., federated learning) amplify functional affordance.

For the social aspect, the system would ideally allow users to share their progress and engage with others, promoting a sense of belonging and support. However, given the scope of this study, this feature was not included in the current implementation. To enhance cognitive affordance, the application offers guidance on the benefits of deep breathing on relaxation and its overall influence on mental health before exercise. This approach strengthens users’ understanding of the intervention’s efficacy and mechanics, potentially boosting its overall effectiveness. Emotional affordance can be stimulated through positive reinforcement and fostering a positive self-perception. The system achieves this by providing encouraging feedback, such as “Good Job” and “Almost there,” following each game attempt. The incremental progress towards the goal and culminating in a blossoming flower upon a successful session establishes a rewarding cycle that promotes emotional satisfaction. Identity affordance is amplified through having a positive self-image through a positive transformation, enhancing both self-efficacy and motivation. Users are invited to embrace this positive identity by envisioning themselves as a dandelion gradually ascending the stairs and ultimately blooming into a flower in the end. This imagery embodies personal growth and positive transformation for the users. Lastly, functional affordance is elevated when users have a clear comprehension of the system’s technical mechanisms, such as federated learning, promoting greater acceptance and trust in the system. We reinforced this by including messages like “synching your breath with the system” during moments of rest in the game.

To sustain user motivation, our system implements progressive disclosure, unveiling features progressively to retain user engagement (30). For instance, the dandelion seed’s ascension up the stairs mirrors the user’s progress.

The efficacy of these design features in fostering engagement, motivating users, and improving the intervention’s effectiveness will be evaluated using appropriate evaluation metrics and methods, which will be elaborated upon in the following sections.

### 3.2 Implementation

Our implementation strategy involved leveraging mobile device technology to create an accessible and effective deep-breathing application. The microphone module of the device is used to detect the user’s breathing, with ambient noise levels measured to ensure accuracy. The MFCC method is utilized for feature extraction, differentiating between correct and incorrect breathing patterns.

During the deep breathing exercises, the system guides users through a series of rest, inhale, and exhale phases, with the dandelion seed’s movement on the screen corresponding to the user’s breathing. We conducted a preliminary study with 40 college students to refine the deep breathing recognition system, creating a dataset of breathing recordings under diverse conditions. This data was used to train and refine our model, ensuring its accuracy and reliability.

The final system was developed using Python and TensorFlow, and the interface was built with Unity. Our approach emphasizes the personalization of the breathing exercises, tailoring the experience to each user’s needs and progress.

The application’s design leverages the built-in microphone of mobile devices to detect users’ breathing patterns, thereby providing gamified biofeedback. As users engage with the system, their continuous breathing practice animates a dandelion seed in the game interface. However, challenges arise when ambient noise might erroneously influence the seed’s movement. Addressing such potential inaccuracies was crucial, as inconsistencies in the feedback can erode users’ trust in the application’s effectiveness (31).

Upon the game’s initiation, the microphone module automatically measures the surrounding noise level for a span of three seconds. This ambient noise reading serves as a baseline against which the user’s breathing is compared with the help of a threshold algorithm. To refine the accuracy of this system, we employed the Mel-Frequency Cepstral Coefficient (MFCC) for feature extraction. Furthermore, convolutional neural network machine-learning techniques were used to discern genuine breathing patterns from both ambient noise and incorrect breathing phases. Figure 4 illustrates the steps of receiving user input, recognizing the breathing phase, collecting newly acquired data, and updating the application’s classification model.

**Figure 2:**
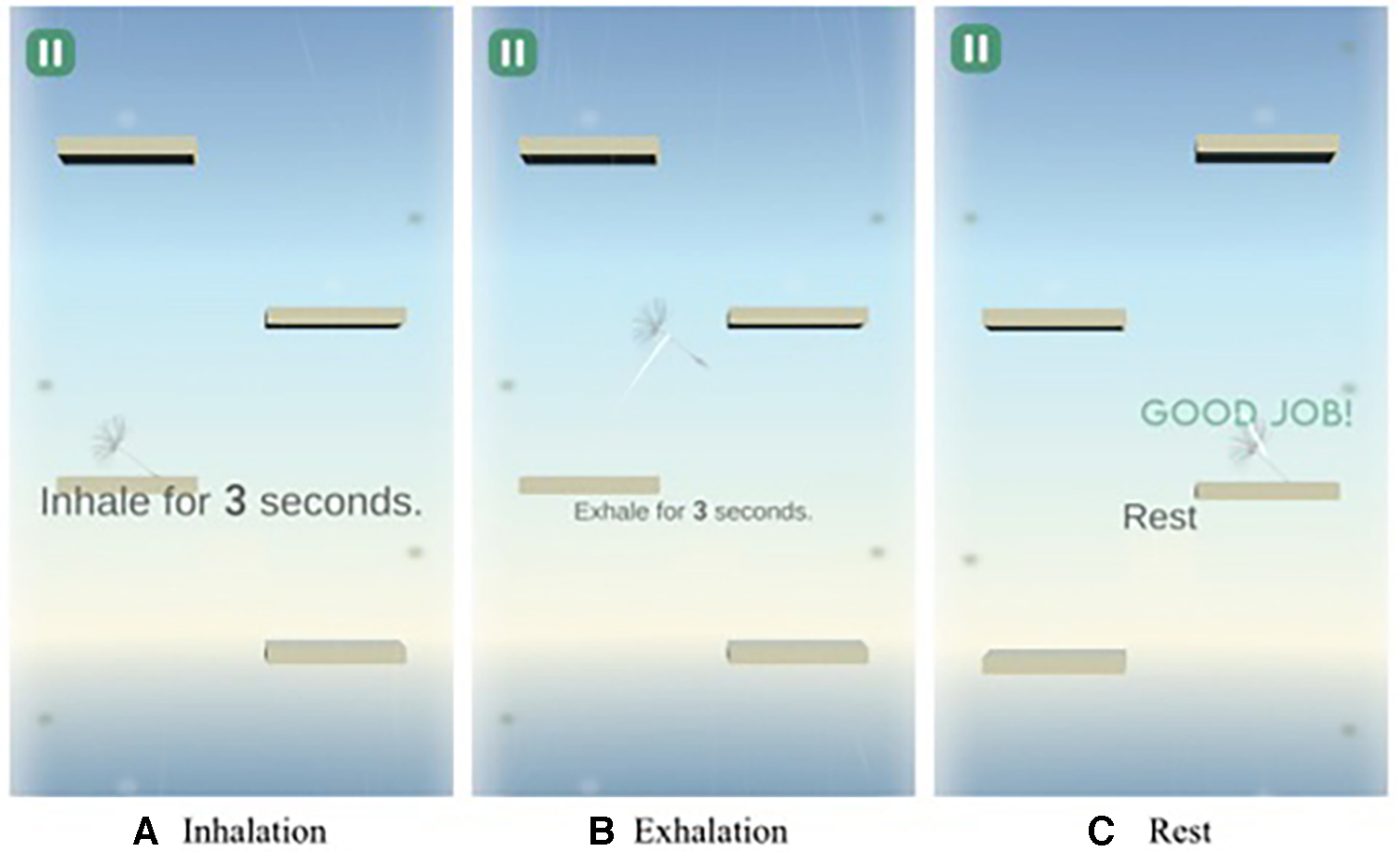
Gamified deep breathing app design.

**Figure 3:**
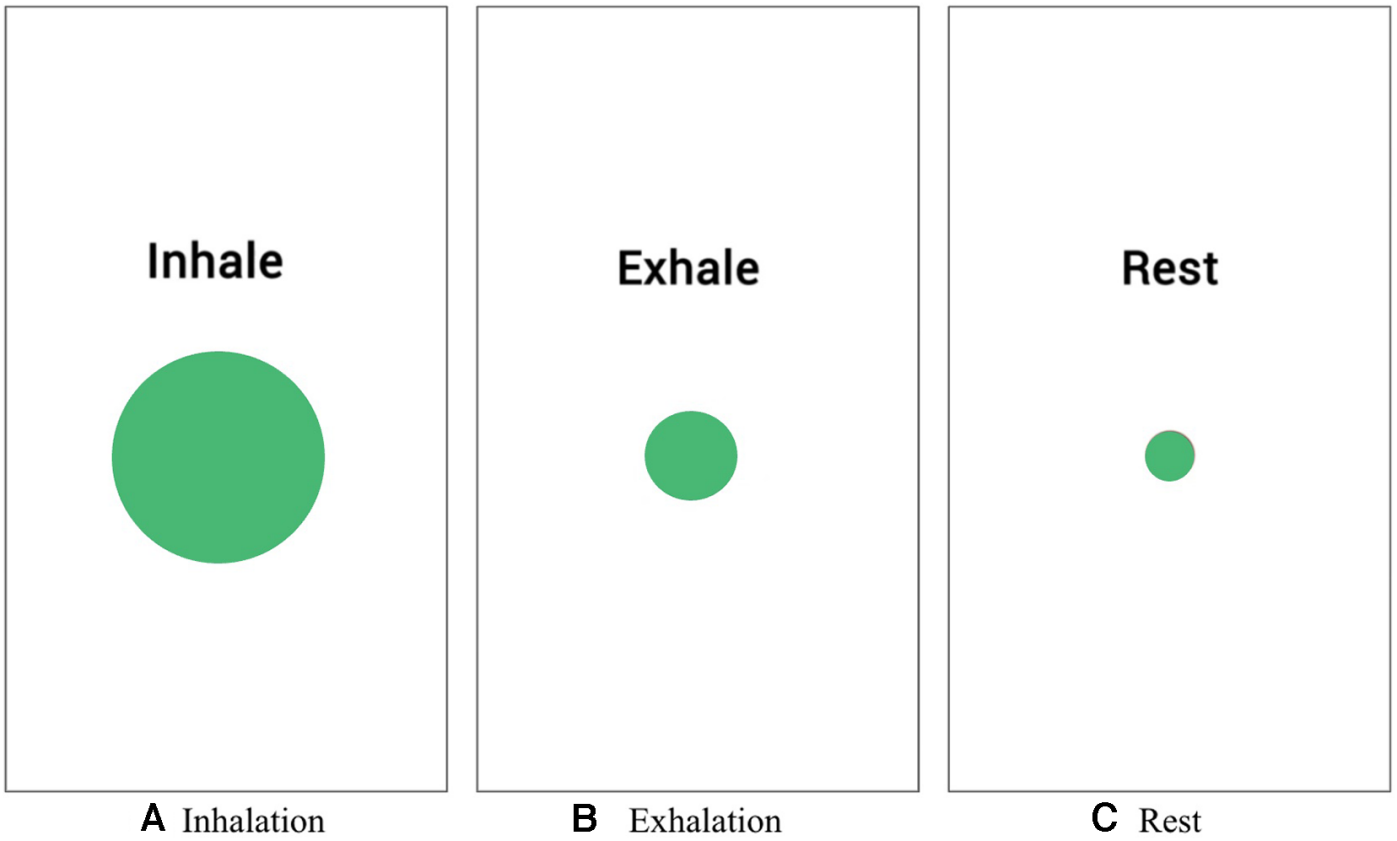
Circle relaxation app design.

**Figure 4:**
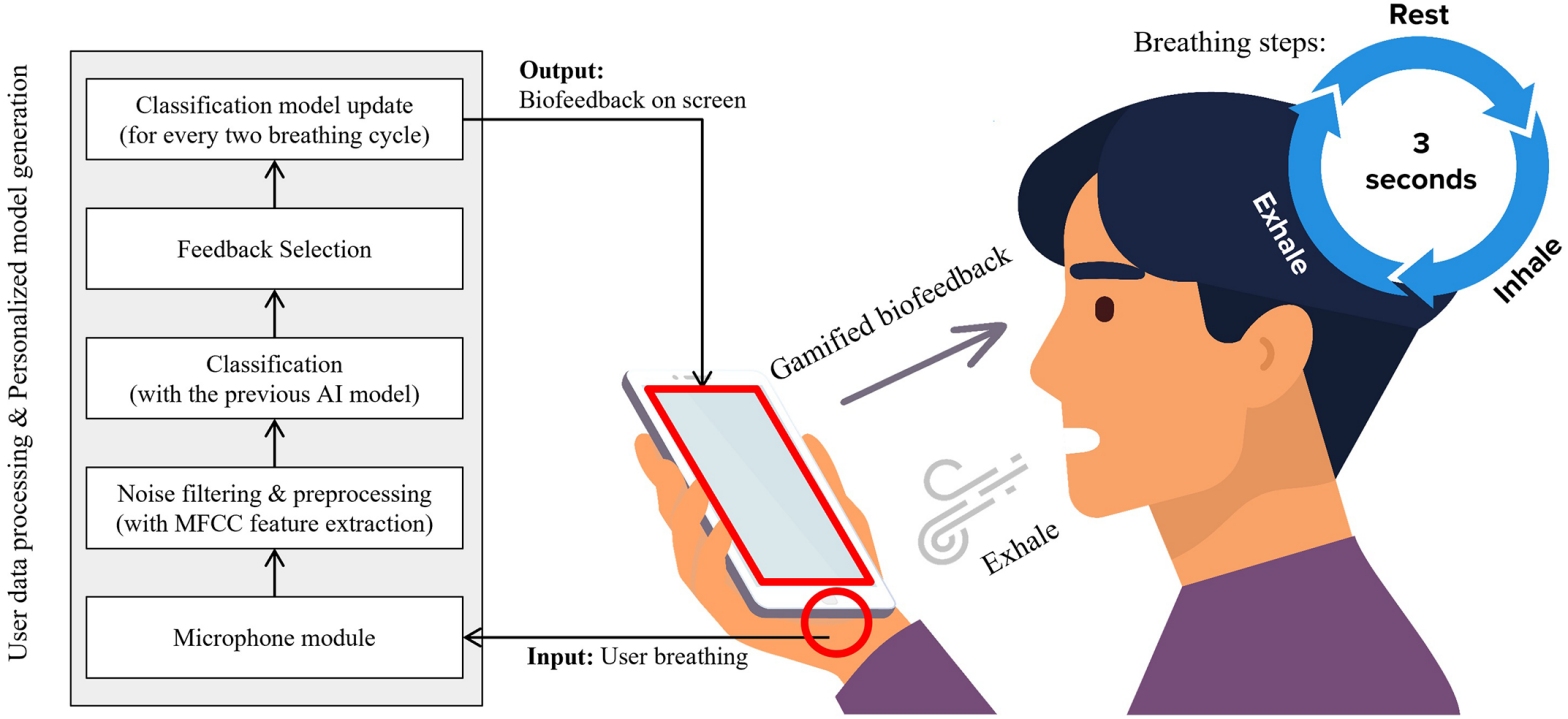
Overview of gamified deep breathing system.

Guided by the application, users cycle through a series of rest, inhale, and exhale phases. During the exhale phase, the application assesses the sound it captures. The dandelion seed’s movement on the screen corresponds to this classified exhalation. A strong exhalation might propel the seed considerably, while a weak one might not move the seed at all. Such visual feedback is instrumental, offering users insights into their breath strength. This dynamic aims not just to mirror the user’s breathing pattern but also to discourage behaviors like hyperventilation and instead foster proper breathing techniques geared towards relaxation.

To refine the deep breathing recognition system, our team conducted a study involving 40 college students. This exercise produced a dataset of a total of 200 1-minute-long breathing recordings made under diverse environmental conditions. Within each recorded file, sections corresponding to exhale, inhale, and rest phase were further subdivided using a windowing method. These recordings were subsequently transformed from their initial time-domain waveform format to a more illustrative mel-spectrogram. The MFCC process was then used to extract distinct sound characteristics from each recording.

The recorded audio signal data, initially in the form of a time-domain waveform, was converted into a mel-spectrogram using a Fourier transform in the frequency spectrum. This conversion enabled us to represent the signal visually. Subsequently, the MFCC method was carried out to extract representative features of each recorded breathing phase (25). Firstly, windowing was applied to divide each crucial section, after which the section energy was computed using the Mel Filter Bank in the power spectrum for each divided signal data (32). By applying cepstral analysis to the Mel spectrum, which was analyzed through the Mel Filter Bank, we could obtain the MFCC. Each audio signal was thus converted into an MFCC format through these processing steps, which facilitated the extraction of unique characteristics of the corresponding sound.

Real-time classification of each breathing phase was made possible by harnessing a MobileNetV2-based model. The lightweight nature of MobileNetV2 made it an apt choice as the foundational architecture for our custom breathing classification model. When our initial model was tested against the MFCC feature data, it boasted an impressive 93.33% accuracy. Moreover, it achieved 93.65% average sensitivity and 96.58% average specificity across the three distinct breathing phases.

**Figure 5:**
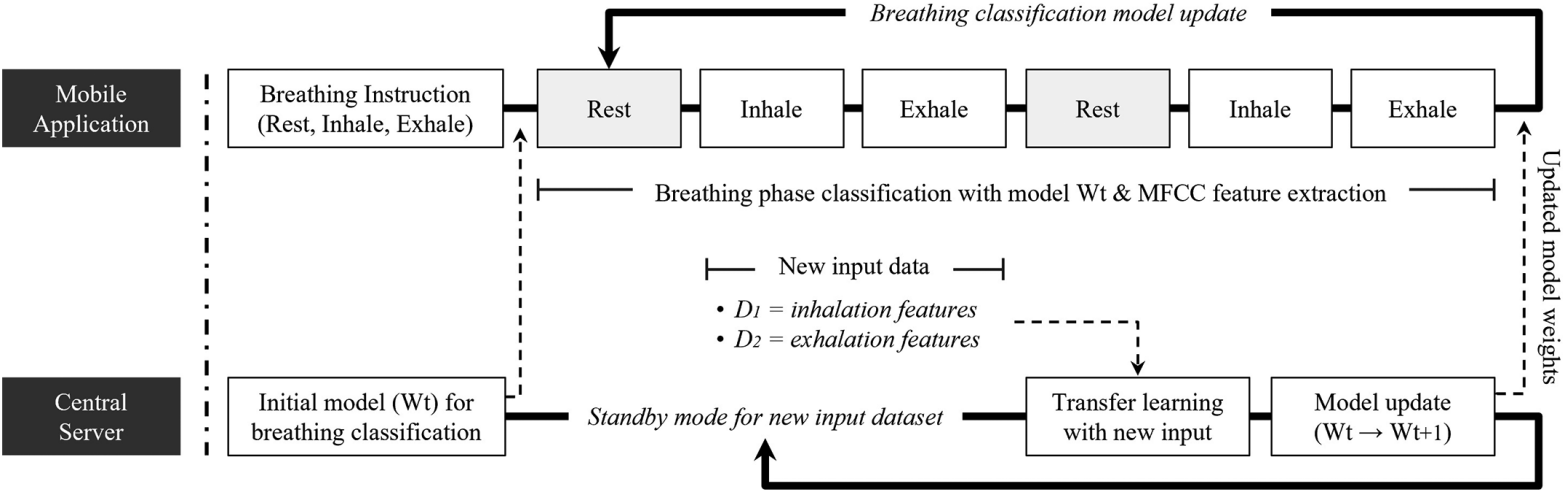
Classification model update process during deep breathing.

A notable aspect of the deep breathing application presented in this study is the personalized classification model update feature for each user. While previous studies also apply transfer learning and machine learning algorithms for user breathing measurement, they implemented a single, generalized weight set model into the system. Contrarily, we designed our proposed breathing application to maximize the effectiveness of digital treatments by enabling each user to cultivate their own optimized breathing model. Figure 5 describes the communication between mobile applications and central servers and how individual models are updated.

As previously mentioned, we adopted and utilized the model update process of the federated learning structure to create and update a model for each user. In a manner similar to federated learning, the initial model Wt is stored on a central server and provided to a new user before the deep breathing system commences. In the mobile application, the method repeats in the order of rest-inhale-exhale and carries out breathing classification and feature extraction using the current model Wt in each section. The newly inputted user breathing data during the inhale and exhale phases are processed, stored, and transmitted to the central server during the next rest cycle. The central server then applies the new input data to the transfer learning algorithm for model weight updates, which are then returned to the mobile application for system updates. A clustering algorithm was applied in the transfer learning process, similar to the technology mentioned in research (33), which helped set a threshold. This threshold allowed individual users to understand the characteristics of each label better. Through this personalized model weight update process, the more each user utilizes the app, the more tailored the model becomes, eventually leading to an optimized breathing threshold.

All these developments and refinements were achieved using Python 3.9.17 and Tensorflow 2.10.1. The final iteration of the system was built with Unity 2021.3.5f1 and optimized for a platform equipped with an Intel Core i7-10700 processor and NVIDIA GeForce RTX3070 graphics.

### 3.3 Experiment setting

To validate the effectiveness and usability of the proposed deep-breathing mobile application, we conducted an experiment with 70 participants. These participants were randomly assigned to either the intervention group, which used our developed system, or the control group, which used an existing deep-breathing mobile application that provides an image to guide the breathing rhythm. The experiment incorporated HRV (Heart Rate Variability) measurements, taken with the ubpulse T1 device developed by LAXTHA, to assess the relaxation effects of deep breathing. Initial baseline HRV measurements were recorded, followed by a one-minute deep breathing session using the respective applications. After the session, HRV measurements were taken again. Following the HRV measurement, participants completed a user experience survey to provide feedback.

Subsequently, our user experience survey was based on the Technology Acceptance Model (TAM). This model emphasizes perceived ease of use and perceived usefulness as key determinants of user attitudes and intentions towards adopting new technologies (34). The survey also incorporated measures of perceived enjoyment, especially considering the gamification elements integrated into our proposed system (35). The primary objective of this survey was to gauge user experiences and their intentions to continue using the deep breathing mobile application over time.

## 4 Results

In our experiment, we enrolled a total of 70 participants. Both the intervention group and the control group consisted of 35 individuals each. The participants had an average age of 33, including 39 females and 31 males. They were meticulously selected, ensuring none had specific heart conditions or were on medications that might influence the Heart Rate Variability (HRV) measurement.

### 4.1 Statistical analysis of HRV metrics

Our study revealed significant changes in the Heart Rate Variability (HRV) indices following the deep breathing exercises. These indices, including normalized low frequency (normLF), normalized high frequency (normHF), Standard Deviation of NN Interval (SDNN), Total Power (TP), and HRV, are pivotal for monitoring the autonomic nervous system’s functionality and assessing resilience to stress.

For the intervention group, the baseline for normLF was (M=53.58, SD=4.44). This value increased to (M=58.10, SD=2.28) during the deep breathing exercises. In tandem with this, the normHF values saw a decrease from its baseline of (M=46.42, SD=4.43) to (M=41.89, SD=2.28) during the exercises.

For the control group, the normLF values increased from a baseline of (M=52.45, SD=5.56) to (M=55.47, SD=2.20) during the exercises. Similarly, normHF values decreased from its baseline (M=47.54, SD=5.56) to (M=44.52, SD=2.20).

The t-test revealed significant p-values for changes in normLF and normHF for both groups, each being *p* 0.05. This indicates that during deep breathing, there’s an increase in sympathetic nerve activity due to lung movement and a corresponding decrease in parasympathetic activity. Notably, the rise in sympathetic nerve activity before and after the deep breathing exercise in the intervention group was significantly pronounced, registering a difference of +4.52 (t=−5.353, p<0.001) in comparison to the control group, which had a difference of +3.02 (t=−2.986, p=0.004). This observation suggests that participants in the intervention group were more engaged, resulting in heightened sympathetic activity (36).

Following the deep breathing exercise, the LF and HF values were measured once again during the recovery phase. The intervention group displayed normLF values of (M=56.22, SD=4.69) and normHF values of (M=43.77, SD=4.69). On the other hand, the control group registered normLF values of (M=54.38, SD=5.28) and normHF values of (M=45.61, SD=5.28). When juxtaposed with the measurements taken during the deep breathing phase, it was evident that both groups exhibited a drop in normLF and a rise in normHF, signaling relaxation.

Delving deeper into the specifics, the intervention group witnessed a statistically significant decline in normLF by −1.88 (t=2.126, p=0.038) and a corresponding increase in normHF by +1.88 (t=−2.126, p=0.038). Conversely, the control group noted a drop in normLF by −1.09 (t=1.13, p=0.264) and an increase in normHF by +1.09 (t=−1.13, p=0.264), but neither of these changes was statistically significant. This indicates that the intervention group, who were more proactive during deep breathing, experienced pronounced activation of the sympathetic nerves and succeeded by activation of the parasympathetic nerves. However, the control group, which was relatively less engaged in deep breathing, did not exhibit a substantial relaxation response afterward.

Subsequent measurements encompassed additional HRV metrics, notably SDNN, TP, and HRV. To discern the significance of these fluctuations, t-test evaluations were executed. For the intervention group, the variations manifested as follows: SDNN had a difference of +24.08 (t=−5.894, p<0.001), TP by +1.07 (t=−5.118, p<0.001), and HRV by +3.91 (t=−3.622, p<0.001), all being statistically significant. In the control group, SDNN changed by +15.98 (t=−3.309, p=0.001), TP by +0.88 (t=−3.702, p<0.001), and HRV by +3.4 (t=−2.664, p=0.009). Though these changes were significant, the scope and amplitude of these modifications were more prominent in the intervention group.

These findings underscore significant shifts in markers of autonomic nervous system activity, notably including the Standard Deviation of the NN Interval (SDNN), TP, and HRV, during deep breathing exercises. SDNN, as a sensitive marker within HRV, plays a pivotal role in indicating an individual’s stress response and overall autonomic nervous system balance (37). A lower SDNN is often associated with reduced stress resilience and poorer mental health outcomes. Thus, the observed reduction in SDNN in our intervention group is particularly meaningful, suggesting that our deep breathing system could be more effective than conventional techniques in enhancing mental health care. These markers not only trace changes in the autonomic nervous system but also reflect an individual’s resilience to stress (38). The more pronounced alterations in the intervention group as opposed to the control group indicate that sustained use of our system might amplify relaxation and bolster stress resilience by positively modulating the autonomic nervous system. This offers valuable support in managing mental health and demonstrates the practical implications of our findings. A detailed breakdown of the HRV measurements is available in Table 1.

**Table 1:** Statistical Analysis result of Intervention group and Control group.

	Frequency domain		Time domain		
	2*norm LF	2*norm HF	2*SDNN	2*TP	2*HRV
Intervention group - Mean (SD)		
Baseline	53.58 (4.44)	46.42 (4.43)	40.57 (18.20)	7.12 (0.92)	11.83 (4.27)
*difference*	+4.52	−4.53	+24.08	+1.07	+3.91
	(p<0.001)	(p<0.001)	(p=1.36e−07)	(p<0.001)	(p<0.001)
Relaxation	58.10 (2.28)	41.89 (2.28)	64.65 (15.90)	8.19 (0.82)	15.74 (4.75)
*difference*	−1.88	+1.88	−27.08	−1.25	−5.31
	(p=0.038)	(p=0.038)	(p<0.001)	(p<0.001)	(p<0.001)
Recovery	56.22 (4.69)	43.77 (4.69)	37.57 (17.46)	6.94 (0.98)	10.43 (3.44)
Control Group - Mean (SD)					
Baseline	52.45 (5.56)	47.54 (5.56)	48.82 (21.76)	7.43 (1.08)	12.90 (5.33)
*difference*	+3.02	−3.02	+15.98	+0.88	+3.40
	(p=0.004)	(p=0.004)	(p=0.001)	(p<0.001)	(p=0.009)
Relaxation	55.47 (2.20)	44.52 (2.20)	64.80 (18.47)	8.31 (0.91)	16.30 (5.33)
*difference*	−1.09	+1.09	−21.16	−1.06	−3.72
	(p=0.264)	(p=0.264)	(p<0.001)	(p<0.001)	(p<0.001)
Recovery	54.38 (5.28)	45.61 (5.28)	43.74 (15.74)	7.25 (0.77)	12.58 (3.95)

Bold indicates significance at *p*-value < 0.05.

### 4.2 Usability analysis

Our analysis extended beyond evaluating the efficacy of the deep breathing system; we delved into its usability as well. A preliminary reliability analysis was performed using Cronbach’s alpha for the gathered results.

The user acceptance of our technology was assessed based on the primary constructs of the Technology Acceptance Model (TAM), specifically: perceived ease of use (PEOU), perceived usefulness (PU), attitude towards using (ATT), and intention to use (ITU). Additionally, we considered perceived enjoyment (PENJ) given its relevance. The reliability metrics for each construct were: PEOU(α=0.86), PU(α=0.73), PENJ(α=0.93), ATT(α=0.83), and ITU(α=0.92). All the Cronbach’s alpha values surpassed the 0.7 threshold, solidifying the reliability of our measures.

Breaking down the results for the intervention group: PEOU scored (M=6.10, SD=0.99), PU at (M=5.41, SD=1.07), PENJ reached (M=5.91, SD=0.98), ATT scored (M=5.80, SD=0.89), and ITU stood at (M=5.19, SD=1.24). In contrast, the control group results were: PEOU (M=6.24, SD=0.97), PU (M=5.28, SD=0.99), PENJ (M=5.00, SD=1.37), ATT (M=5.24, SD=1.18), and ITU (M=4.87, SD=1.70). Interestingly, aside from PEOU, all metrics indicated a preference for the intervention system.

**Figure 6:**
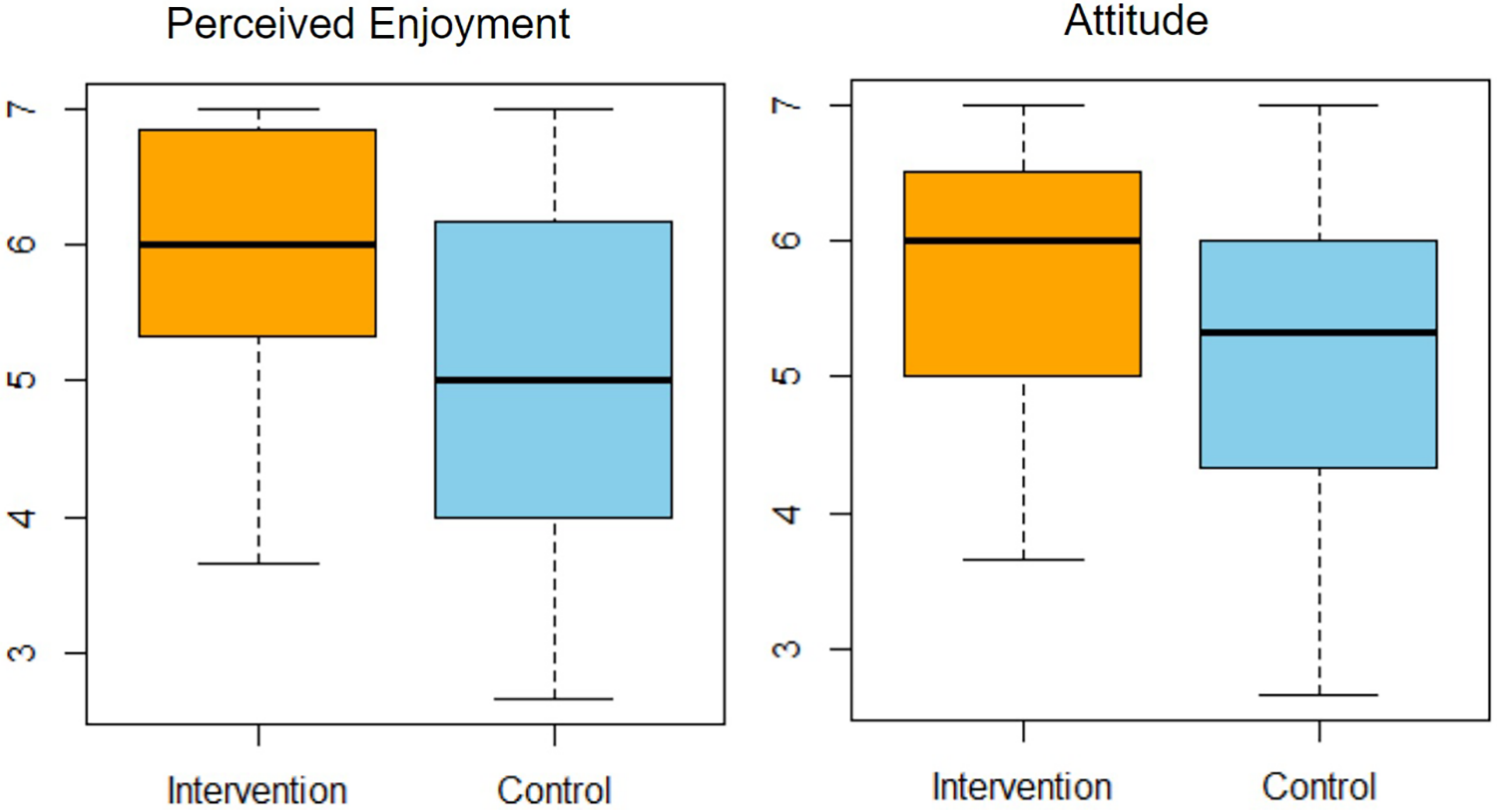
Boxplot of perceived enjoyment and attitude.

In the subsequent t-test, only PENJ (t=3.175, p=0.002) and ATT (t=2.237, p=0.028) showcased significant disparities between the groups. However, when introducing a regression analysis, considering ITU as the dependent variable, both PENJ (t=7.743, p<0.001) and ATT (t=7.516, p<0.001) emerged significantly. This suggests that PENJ and ATT might play pivotal roles in determining the long-term inclination towards our deep breathing system. These findings are graphically represented in Figure 6.

## 5 Discussion

In summarizing our findings regarding the efficacy and usability of our evolving deep breathing system in the realm of digital healthcare and Digital Therapeutics for mental well-being, our study illuminates several key aspects and future potentials.

The integration of gamification in our system, characterized by elements such as enjoyment and interactive feedback, has significantly improved participant commitment to deep breathing exercises. Furthermore, the application of machine learning, particularly the use of Mel Frequency Cepstral Coefficient (MFCC) for real-time assessment and feedback of breathing states, has shown promising results in enhancing the effectiveness of our deep breathing exercises. Our results showed his technological advancement is pivotal in creating a more personalized and responsive therapeutic experience.

Moreover, our study’s utilization of Heart Rate Variability (HRV) as a dynamic indicator of the autonomic nervous system’s functionality has provided invaluable insights. The analysis of HRV data, through frequency and time domain methods, has helped us understand the physiological impact of our deep breathing exercises on mental well-being. This comprehensive measurement approach underpins the potential effectiveness of our interventions in managing mental health conditions like anxiety, stress, and mood disorders.

Looking towards the future, we envision our system’s continued refinement leading to more widespread and effective use in DTx applications. Its ability to actively engage the autonomic nervous system suggests that it could be applied to a broader range of mental health conditions, potentially offering a non-invasive, user-friendly alternative to more traditional therapies (39). The observed patterns in Low Frequency (LF) and High Frequency (HF) values during exercises indicate a direct impact on the autonomic nervous system, providing a basis for exploring its application in managing conditions like anxiety, stress, and mood disorders (40).

In terms of usability, our system’s positive reception compared to traditional deep breathing exercises positions it as a more engaging and effective tool for mental health management. This user-friendly approach, coupled with the potential for sustained use, underlines our system’s capacity to deliver long-term therapeutic benefits (41). It also opens avenues for integration with other digital health applications, enhancing overall mental health care efficacy (42).

Overall, the implications of our research extend beyond immediate findings, suggesting a transformative potential for digital therapeutics in mental health management, both now and in the future.

### 5.1 Limitation & future works

The present study mainly pivoted towards optimizing for mobile platforms, mirroring the digital health industry’s trajectory. Consequently, it might not have fully capitalized on the potential of existing computer technology. For instance, our study employed a model updating framework akin to federated learning for personalizing the classification model. However, inherent limitations in mobile-based self-learning prompted us to rely on a method that sends user data to a central server. This aspect highlights the necessity for subsequent research, especially as mobile technologies continue to evolve. Moreover, our system design didn’t manage to incorporate every suggested design element derived from an affordance-based standpoint, primarily because our focus was on initial validation in the absence of any affordance-based designed breathing systems. We foresee more refined research addressing these gaps. Finally, while the gender and age distribution of our participants provided initial insights, the limited sample size of 70 may constrain the generalizability of our findings. Future studies with larger and more diverse samples are essential to validate and extend our findings.

## 6 Conclusion

In our research, we introduced and assessed a deep breathing system tailored for digital healthcare and Digital Therapeutics contexts. Our findings underscore the system’s potential to substantially enhance mental health by fostering engaging and personalized user interactions. The integration of gamification and machine learning-driven personalized feedback has successfully motivated users to be more proactive during the deep breathing exercises. This heightened involvement manifests physiologically, evidenced by the rise in LF values during exercises, followed by an increase in HF, signifying exertion and subsequent relaxation effects. Moreover, the data suggest that our approach can invigorate the autonomic nervous system, potentially boosting stress resilience and further emphasizing the therapeutic promise of our solution.

From a usability perspective, participants expressed a marked preference for our system over traditional deep breathing alternatives, recounting a more delightful and affirmative experience. This enthusiasm is a robust testament to the system’s potential for consistent use, a crucial aspect for realizing enduring therapeutic advantages.

Despite these positive strides, the study illuminated avenues for refinement and future exploration. Prioritizing the full spectrum of computer technology, embedding more affordance-driven design facets, and tapping into a wider participant demographic emerged as the study’s limitations. These identified gaps are envisioned to steer forthcoming advancements and scholarly pursuits, paving the way for even more sophisticated digital therapeutic instruments dedicated to mental wellness.

In summation, our innovative deep breathing system represents a seminal advancement in digital healthcare. It not only acts as a potent stress alleviation tool but also cultivates a culture of active user participation, leading to enhanced mental well-being. The challenges and prospective research pathways spotlighted in our study offer exciting prospects for refinement and breakthroughs in this swiftly progressing sector.

## Data Availability

The datasets for this article are not publicly available due to concerns regarding participant anonymity. Requests to access the datasets should be directed to the corresponding author.

## References

[1] Torous J Myrick KJ Rauseo-Ricupero N Firth J. Digital mental health, COVID-19: using technology today to accelerate the curve on access, quality tomorrow. JMIR Ment Health. (2020) 7:e18848. 10.2196/18848 32213476 PMC7101061

[2] Cho C-H Lee H-J. Could digital therapeutics be a game changer in psychiatry?. Psychiatry Investig. (2019) 16:97. 10.30773/pi.2019.01.20 30808114 PMC6393746

[3] Hong JS Wasden C Han DH. Introduction of digital therapeutics. Comput Methods Programs Biomed. (2021) 209:106319. 10.1016/j.cmpb.2021.106319 34364181

[4] Kadakia K Patel B Shah A. Advancing digital health: FDA innovation during COVID-19. Npj Digit Med. (2020) 3:1–3. 10.1038/s41746-020-00371-7 33335250 PMC7747714

[5] Zhu B Hedman A Feng S Li H Osika W. Designing, prototyping and evaluating digital mindfulness applications: a case study of mindful breathing for stress reduction. J Med Internet Res. (2017) 19:e197. 10.2196/jmir.6955 28615157 PMC5489711

[6] Chien H-C Chung Y-C Yeh M-L Lee J-F. Breathing exercise combined with cognitive behavioural intervention improves sleep quality and heart rate variability in major depression. J Clin Nurs. (2015) 24:3206–14. 10.1111/jocn.12972 26404039

[7] Alsawaier RS. The effect of gamification on motivation and engagement. Int J Inf Learn Technol. (2018) 35(1):56–79.

[8] Sardi L Idri A Fernández-Alemán JL. A systematic review of gamification in e-Health. J Biomed Inform. (2017) 71:31–48. 10.1016/j.jbi.2017.05.011 28536062

[9] Oyebode O Fowles J Steeves D Orji R. Machine learning techniques in adaptive, personalized systems for health, wellness. Int J Hum Comput Interact. (2023) 39(9):1938–62.

[10] Khaddage F Lattemann C Acosta-Díaz R. Mobile gamification in education engage, educate, entertain via gamified mobile apps. In: *Society for Information Technology & Teacher Education International Conference*. Association for the Advancement of Computing in Education (AACE) (2014). p. 1654–60.

[11] Stepanovic S Mettler T. Gamification applied for health promotion: does it really foster long-term engagement? a scoping review. In: *Proceedings of the 26th European Conference on Information Systems*. AIS (2018). p. 1–6.

[12] Hammedi W Leclerq T Van Riel AC. The use of gamification mechanics to increase employee, user engagement in participative healthcare services: a study of two cases. J Serv Manag. (2017) 28:640–61. 10.1108/JOSM-04-2016-0116

[13] Cugelman B. Gamification: what it is and why it matters to digital health behavior change developers. JMIR Ser Games. (2013) 1:e3139. 10.2196/games.3139 PMC4307817 25658754

[14] Brown M O’Neill N van Woerden H Eslambolchilar P Jones M John A. et al. Gamification, adherence to web-based mental health interventions: a systematic review. JMIR Ment Health. (2016) 3:e5710. 10.2196/mental.5710 PMC5014987 27558893

[15] Agrawal V Naik V Duggirala M Athavale S. Calm a mobile based deep breathing game with biofeedback. In: *Extended Abstracts of the 2020 Annual Symposium on Computer–Human Interaction in Play* (2020). p. 153–7.

[16] Lukic YX Teepe GW Fleisch E Kowatsch T. Breathing as an input modality in a gameful breathing training app (breeze 2): development, evaluation study. JMIR Ser Games. (2022) 10:e39186. 10.2196/39186 PMC9428773 35972793

[17] Shih C-H Tomita N Lukic YX Kowatsch T. Breeze: smartphone-based acoustic real-time detection of breathing phases for a gamified biofeedback breathing training. Proc ACM Interact Mob Wearable Ubiquitous Technol. (2019) 3:1–30. 10.1145/336983534164595

[18] Lupton D. The digitally engaged patient: self-monitoring and self-care in the digital health era. Soc Theory Health. (2013) 11:256–70. 10.1057/sth.2013.10

[19] Yoo JH Jeong H Chung T-M. Cutting-edge technologies for digital therapeutics: a review and architecture proposals for future directions. Appl Sci. (2023) 13:6929. 10.3390/app13126929

[20] Masoudi N Fadel GM Pagano CC Elena MV. A review of affordances and affordance-based design to address usability. In: *Proceedings of the Design Society: International Conference on Engineering Design*. Cambridge University Press, vol. 1 (2019). p. 1353–62.

[21] Wong CA Madanay F Ozer EM Harris SK Moore M Master SO. et al. Digital health technology to enhance adolescent and young adult clinical preventive services: affordances and challenges. J Adolesc Health. (2020) 67:S24–S33. 10.1016/j.jadohealth.2019.10.018 32718511 PMC11925059

[22] Cho Y Bianchi-Berthouze N Julier SJ. Deepbreath: deep learning of breathing patterns for automatic stress recognition using low-cost thermal imaging in unconstrained settings. In: *2017 Seventh International Conference on Affective Computing and Intelligent Interaction (ACII)*. IEEE (2017). p. 456–63.

[23] Cochrane KA Loke L Ahmadpour N Schiphorst T Campbell A Núñez-Pacheco C. A comparison design study of feedback modalities to support deep breathing whilst performing work tasks. Work. (2021) 68:1187–202. 10.3233/WOR-213448 33867378

[24] Han W Chan C-F Choy C-S Pun K-P. An efficient MFCC extraction method in speech recognition. In: *2006 IEEE International Symposium on Circuits and Systems (ISCAS)*. IEEE (2006). p. 4.

[25] Hossan MA Memon S Gregory MA. A novel approach for MFCC feature extraction. In: *2010 4th International Conference on Signal Processing and Communication Systems*. IEEE (2010). p. 1–5.

[26] Sadi TM Hassan R. Development of classification methods for wheeze and crackle using mel frequency cepstral coefficient (MFCC): A deep learning approach. Int J Percept Cogn Comput. (2020) 6:107–14. 10.31436/ijpcc.v6i2.166

[27] Kemp AH Quintana DS. The relationship between mental and physical health: insights from the study of heart rate variability. Int J Psychophysiol. (2013) 89:288–96. 10.1016/j.ijpsycho.2013.06.018 23797149

[28] Appelhans BM Luecken LJ. Heart rate variability as an index of regulated emotional responding. Rev Gen Psychol. (2006) 10:229–40. 10.1037/1089-2680.10.3.229

[29] Prinsloo GE Derman WE Lambert MI Laurie Rauch H. The effect of a single session of short duration biofeedback-induced deep breathing on measures of heart rate variability during laboratory-induced cognitive stress: a pilot study. Appl Psychophysiol Biofeedback. (2013) 38:81–90. 10.1007/s10484-013-9210-0 23435801

[30] Springer A Whittaker S. Progressive disclosure: empirically motivated approaches to designing effective transparency. In: *Proceedings of the 24th International Conference on Intelligent User Interfaces* (2019). p. 107–20.

[31] Yu K Berkovsky S Taib R Conway D Zhou J Chen F. User trust dynamics: an investigation driven by differences in system performance. In: *Proceedings of the 22nd International Conference on Intelligent User Interfaces* (2017). p. 307–17.

[32] Madikeri SR Murthy HA. Mel filter bank energy-based slope feature and its application to speaker recognition. In: *2011 National Conference on Communications (NCC)*. IEEE (2011). p. 1–4.

[33] Yoo JH Son HM Jeong H Jang E-H Kim AY Yu HY. et al. Personalized federated learning with clustering: non-iid heart rate variability data application. In: *2021 International Conference on Information and Communication Technology Convergence (ICTC)*. IEEE (2021). p. 1046–51.

[34] Wallace LG Sheetz SD. The adoption of software measures: A technology acceptance model (TAM) perspective. Inf Manag. (2014) 51:249–59. 10.1016/j.im.2013.12.003

[35] Hussain A Mkpojiogu EO Yusof MM. Perceived usefulness, perceived ease of use, and perceived enjoyment as drivers for the user acceptance of interactive mobile maps. In: *AIP Conference Proceedings*. AIP Publishing, vol. 1761 (2016).

[36] Reyes del Paso GA Langewitz W Mulder LJ Van Roon A Duschek S. The utility of low frequency heart rate variability as an index of sympathetic cardiac tone: a review with emphasis on a reanalysis of previous studies. Psychophysiology. (2013) 50:477–87. 10.1111/psyp.12027 23445494

[37] Kim H-G Cheon E-J Bai D-S Lee YH Koo B-H. Stress and heart rate variability: a meta-analysis and review of the literature. Psychiatry Investig. (2018) 15:235. 10.30773/pi.2017.08.17 29486547 PMC5900369

[38] Jarczok MN Jarczok M Mauss D Koenig J Li J Herr RM. et al. Autonomic nervous system activity and workplace stressors—a systematic review. Neurosci Biobehav Rev. (2013) 37:1810–23. 10.1016/j.neubiorev.2013.07.004 23891906

[39] D’Ostilio K Magis D. Invasive and non-invasive electrical pericranial nerve stimulation for the treatment of chronic primary headaches. Curr Pain Headache Rep. (2016) 20:1–9. 10.1007/s11916-016-0589-1 27678260

[40] Nakajima Y Tanaka N Mima T Izumi S-I. Stress recovery effects of high-and low-frequency amplified music on heart rate variability. Behav Neurol. (2016) 2016:1–9. 10.1155/2016/5965894 PMC5021883 27660396

[41] Bickmore TW Picard RW. Establishing and maintaining long-term human-computer relationships. ACM Trans Comput Hum Interact. (2005) 12:293–327. 10.1145/1067860.1067867

[42] Dang A Arora D Rane P. Role of digital therapeutics and the changing future of healthcare. J Family Med Prim Care. (2020) 9:2207. 10.4103/jfmpc.jfmpc-105-20 32754475 PMC7380804

